# Enhancement of astaxanthin production in *Xanthophyllomyces dendrorhous* by efficient method for the complete deletion of genes

**DOI:** 10.1186/s12934-016-0556-x

**Published:** 2016-09-13

**Authors:** Keisuke Yamamoto, Kiyotaka Y. Hara, Toshihiko Morita, Akira Nishimura, Daisuke Sasaki, Jun Ishii, Chiaki Ogino, Noriyuki Kizaki, Akihiko Kondo

**Affiliations:** 1 Department of Chemical Science and Engineering, Graduate School of Engineering, Kobe University, 1-1 Rokkodaicho, Nada-ku, Kobe, 657-8501 Japan; 2 Division of Environmental and Life Sciences, Graduate Division of Nutritional and Environmental Sciences, University of Shizuoka, 52-1 Yada, Suruga-ku, Shizuoka 422-8526 Japan; 3 Organization of Advanced Science and Technology, Kobe University, 1-1 Rokkodaicho, Nada-ku, Kobe, 657-8501 Japan; 4 Medical Device Development Laboratories, Kaneka Corporation, Takasago, Hyogo Japan; 5 Graduate School of Science, Technology, and Innovation, Kobe University, 1-1 Rokkodai-cho, Nada-ku, Kobe, Hyogo 657-8501 Japan; 6 Biotechnology Development Laboratories, Kaneka Corporation, Takasago, Hyogo Japan

**Keywords:** Gene deletion, Astaxanthin production, Yeast, *Xanthophyllomyces dendrorhous*, Metabolic engineering

## Abstract

**Background:**

Red yeast, *Xanthophyllomyces dendrorhous* is the only yeast known to produce astaxanthin, an anti-oxidant isoprenoid (carotenoid) widely used in the aquaculture, food, pharmaceutical and cosmetic industries. The potential of this microorganism as a platform cell factory for isoprenoid production has been recognized because of high flux through its native terpene pathway. Recently, we developed a multiple gene expression system in *X. dendrorhous* and enhanced the mevalonate synthetic pathway to increase astaxanthin production. In contrast, the mevalonate synthetic pathway is suppressed by ergosterol through feedback inhibition. Therefore, releasing the mevalonate synthetic pathway from this inhibition through the deletion of genes involved in ergosterol synthesis is a promising strategy to improve isoprenoid production. An efficient method for deleting diploid genes in *X*. *dendrorhous*, however, has not yet been developed.

**Results:**

*Xanthophyllomyces dendrorhous* was cultivated under gradually increasing concentrations of antibiotics following the introduction of antibiotic resistant genes to be replaced with target genes. Using this method, double *CYP61* genes encoding C-22 sterol desaturases relating to ergosterol biosynthesis were deleted sequentially. This double *CYP61* deleted strain showed decreased ergosterol biosynthesis compared with the parental strain and single *CYP61* disrupted strain. Additionally, this double deletion of *CYP61* genes showed increased astaxanthin production compared with the parental strain and the single *CYP61* knockout strain. Finally, astaxanthin production was enhanced by 1.4-fold compared with the parental strain, although astaxanthin production was not affected in the single *CYP61* knockout strain.

**Conclusions:**

In this study, we developed a system to completely delete target diploid genes in *X. dendrorhous*. Using this method, we deleted diploid *CYP61* genes involved in the synthesis of ergosterol that inhibits the pathway for mevalonate, which is a common substrate for isoprenoid biosynthesis. The resulting decrease in ergosterol biosynthesis increased astaxanthin production. The efficient method for deleting diploid genes developed in this study has the potential to improve industrial production of various isoprenoids in *X. dendrorhous*.

**Electronic supplementary material:**

The online version of this article (doi:10.1186/s12934-016-0556-x) contains supplementary material, which is available to authorized users.

## Background

Carotenoids are widely distributed in nature and are exclusively synthesized by plants and microorganisms [1]. Carotenoids belong to the natural compounds class of terpenes (isoprenoids) [2]. Bioproduction of pharmaceutically important carotenoids such as artemisinin and Taxol precursor, has been accomplished by genetically engineered, well-characterized microorganisms such as *Saccharomyces cerevisiae* and *Escherichia coli* [3, 4]. Recently, however, Melillo, et al. [5] showed the potential of red yeast, *Phaffia rhodozyma* (sexual form, *Xanthophyllomyces dendrorhous*), as a platform microorganism for isoprenoid production because of the higher rates of flux through its native terpene pathway compared with *S. cerevisiae* and *E. coli*. *X. dendrorhous* has been studied as a promising candidate microorganism for maximizing production of the carotenoid astaxanthin (3, 3′-dihydroxy-β, β-carotene-4, 4′-dione; C_40_H_52_O_4_). Astaxanthin is used in fine chemical industries such as the food, pharmaceutical, and cosmetics industries because of its antioxidant properties [6, 7].

We previously identified strong promoters for the expression of target genes and developed a multi-gene expression system in *X. dendrorhous* [7, 8]. Astaxanthin production was enhanced by metabolic engineering using this system [8]. In contrast, high astaxanthin-producing mutant strains were previously obtained from randomly mutated strains [9, 10]. In most of these strains, the critical mutations for enhancing astaxanthin biosynthesis have not been identified; however, these most likely involve a lack of genes affecting astaxanthin biosynthesis. Thus, gene disruption was a very promising strategy to enhance astaxanthin production. However, a problem with random mutagenesis is the potential occurrence of unexpected mutations that could have negative effects on industrial astaxanthin production. Indeed, though astaxanthin content was increased by random mutagenesis, cell growth was inhibited [10]. A decrease in the final cell concentration has a negative effect on the volumetric concentration of astaxanthin, the most important factor for industrial astaxanthin production. Thus, a targeted gene disruption technique is required for deleting genes that negatively affect astaxanthin production. A gene deletion technique is essential for further improvement of astaxanthin production in *X. dendrorhous*. Two methods for the deletion of target genes in *X. dendrorhous* have been reported previously. Mauricio et al. [11] reported the double recombinant method in 2008, where two different concentrations of antibiotics were used. Some researchers, including the authors, tried and failed to delete target genes using the double recombinant method [12, 13]. In contrast, Iris et al. [14] reported a method to delete target diploid genes using two different antibiotic resistant genes. This method can be used for deletion of only two diploid genes because only four antibiotic-resistant genes have been known to work as antibiotic selection markers in *X. dendrorhous*.

**Fig. 1:**
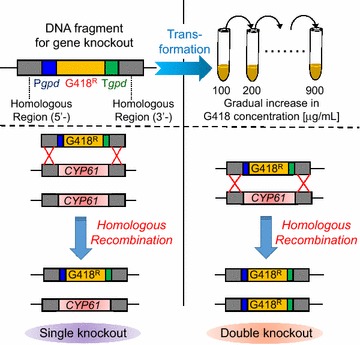
The outline of a novel method for double deletion of diploid target genes in *X. dendrorhous* using one drug-resistant gene

**Fig. 2:**
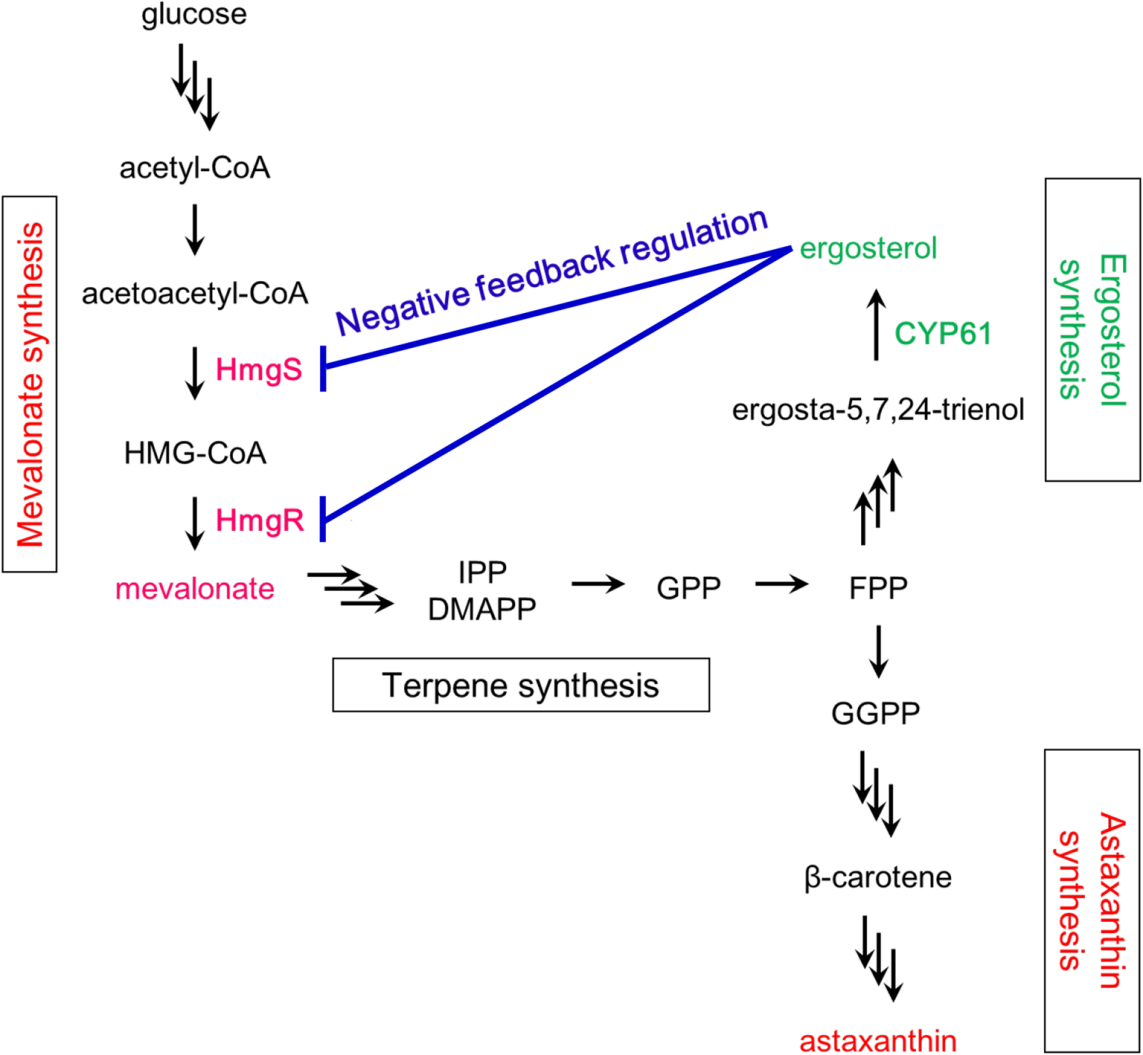
Astaxanthin biosynthesis and negative feedback regulation by ergosterol in *X. dendrorhous*. Astaxanthin is biosynthesized from acetyl-CoA through the pathway consists of the mevalonate, terpene, β-carotene and astaxanthin biosynthetic pathways. Astaxanthin biosynthesis was inhibited through negative feedback regulation by ergosterol synthesized by *CYP61* gene product. *IPP* isopentenyl-pyrophosphate, *DMAPP* dimethylallyl-pyrophosphate, *GPP* geranyl-pyrophosphate, *FPP* farnesyl-pyrophosphate, *GGPP* geranylgeranyl-pyrophosphate, *HMGS* HMG-CoA synthase, *HmgR* HMG-CoA reductase, *CrtE* geranylgeranyl-pyrophosphate synthase, *CrtS* astaxanthin synthase. IPP and DMAPP are the isoprenoid building blocks and prenyltransferases generate GPP, FPP and GGPP, the common precursors of monoterpenoids (C10), sesquiterpenoids (C15) and diterpenoids (C20), respectively. Carotenoids (C40) such as astaxanthin are constructed by condensation of two molecules of GGPP

In this study, we developed an easy and novel method for the double deletion of diploid target genes in *X. dendrorhous* using one antibiotic resistant gene that saves selection markers. The outline of this new method is shown in Fig. 1. Using this method, we deleted both diploid *CYP61* genes encoding C-22 sterol desaturases, which negatively regulate hydroxymethylglutaryl-coenzyme A (HMG-CoA) synthase (HmgS) and HMG-CoA reductase (HmgR) in the ergosterol biosynthesis, the rate-limiting enzymes in the mevalonate synthetic pathway [14] (Fig. 2). The double deletion of diploid *CYP61* genes decreased the ergosterol biosynthesis, leading to improved astaxanthin production.

## Results and discussion

### An efficient method for double deletion of target genes in *X. dendrorhous*

We developed an efficient method for double deletion of target diploid genes in *X. dendrorhous* through a single genetic transformation event followed by cultivation with gradually increasing concentrations of antibiotics. This study targeted the diploid *CYP61* genes for deletion to develop the method. It was expected that double deletion of *CYP61* genes would suppress synthesis of ergosterol and, therefore, increase astaxanthin content by releasing the mevalonate biosynthesis pathway from feedback inhibition by ergosterol.

**Fig. 3:**
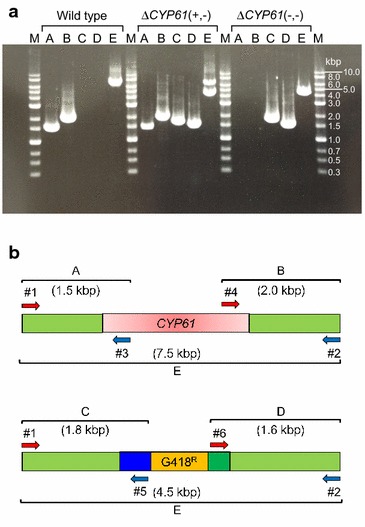
PCR-based analysis of *CYP61* mutants. **a** The diagram shows the amplification target and each set of primers. **b** The gel shows PCR reactions performed with different sets of primers and genomic DNA from the parental strain (WT), Δ*CYP61*(+, −) strain and Δ*CYP61*(−, −) strain. *Lanes A*, *B*, *C*, *D* and *E* represent the fragment between primers #1 and #3 (1.5 kbp), #2 and #4 (2.0 kbp), #1 and #5 (1.8 kbp), #2 and #6 (1.6 kbp), and #1 and #2 (7.5 kbp for *CYP61* gene and 4.5 kbp for G418^R^ gene), respectively. The estimated sizes of the PCR fragments were represented in parenthesis. *Lane M* represent DNA marker and *numbers* on the *right side* show molecular weights of DNA marker

Firstly, the plasmid pKF-G418-g*CYP61* digested with *NdeI* was transformed into the parental *X. dendrorhous* host strain to construct the *CYP61* single disruption strain [Δ*CYP61*(+, −)]. The genotype of the Δ*CYP61*(+, −) strain was confirmed by PCR using specific primers for the *CYP61* gene and G418 resistant cassette (Fig. 3a, b). Specific fragments of *CYP61* (A and B) were confirmed in both the parental host strain and Δ*CYP61*(+, −) strain. Conversely, the specific fragments for the G418 resistant cassette (C and D) were confirmed in the Δ*CYP61*(+, −) strain, but not in the parental host strain. Furthermore, the theoretical length of fragment E from the G418 resistant cassette (4.5 kbp) appeared in the Δ*CYP61*(+, −) strain, while the *CYP61* gene fragment (7.5 kbp) appeared in both the parental host strain and the Δ*CYP61*(+, −) strain. These results indicated that a single *CYP61* gene was disrupted in the Δ*CYP61*(+, −) strain. This first disruption of a single target gene would be replaced using a genome editing technology such as the CRISPR-Cas9 system.

**Fig. 4:**
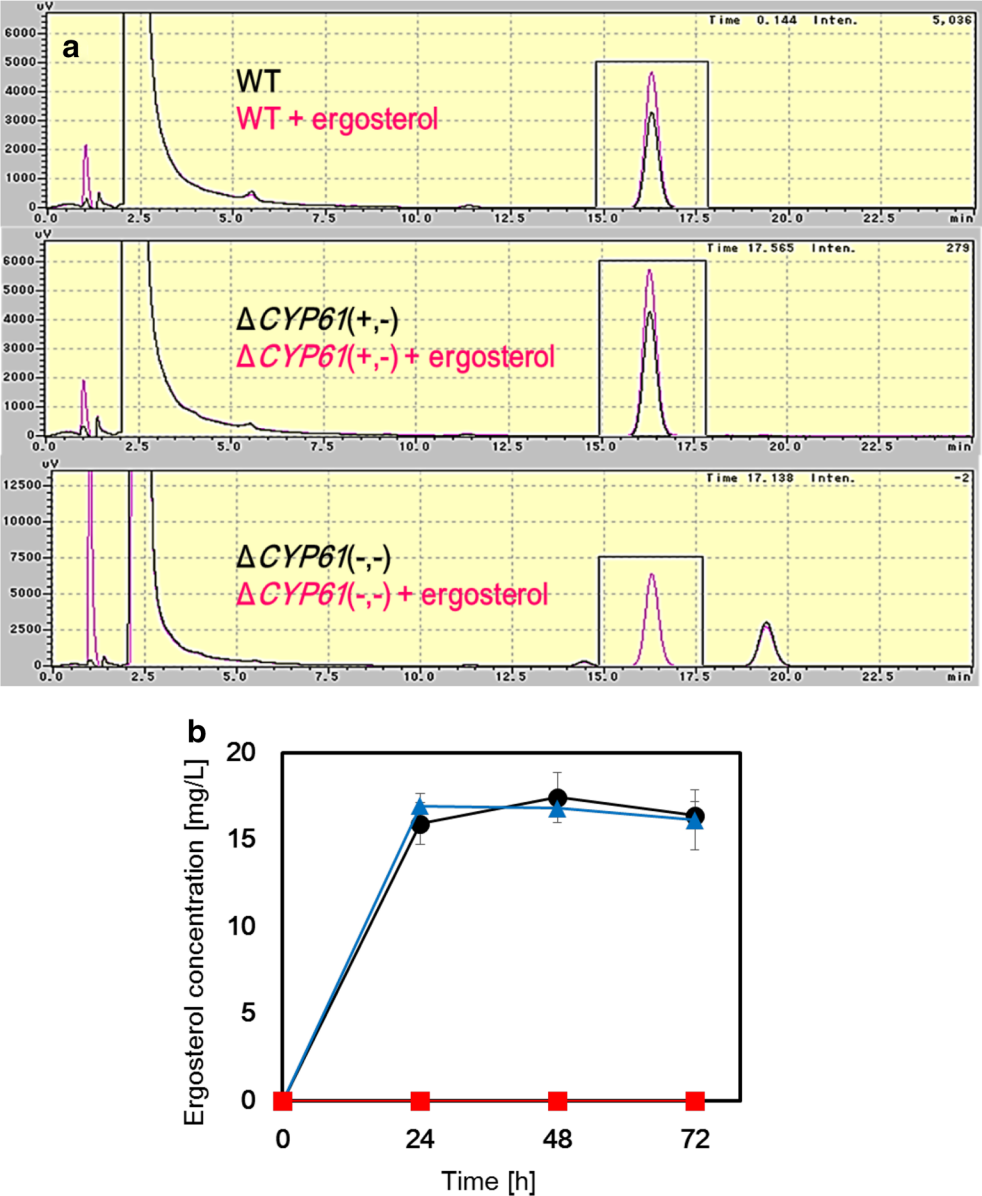
Ergosterol biosynthesis. **a** RP-HPLC ergosterol analysis from the parental host strain, Δ*CYP61*(+, −) and Δ*CYP61*(−, −). Chromatograms (at 280 nm) correspond to ergosterol extracted from strains as described in the “Methods” section. Ergosterol extracts (*black*) from the parental host strain, Δ*CYP61*(+, −) strain and Δ*CYP61*(−, −) strain were analyzed with co-injection of ergosterol standard (*pink*). **b** Ergosterol production in the parental host strain, Δ*CYP61*(+, −) strain and Δ*CYP61*(−, −) strain. *Circle* (*black*), *triangle* (*blue*) and *square* (*red*) symbols represent values of the parental host strain, Δ*CYP61*(+, −) strain and Δ*CYP61*(−, −) strain, respectively. The values are means and the *error bars* show the standard deviation (*n* = 3)

Next, we carried out disruption of both *CYP61* genes because ergosterol biosynthesis of Δ*CYP61*(+, −) strain was almost equal to that of the parental strain as shown in Fig. 4 and described in detail in the following section “Ergosterol biosynthesis”. The sequential operations were carried out to cultivate Δ*CYP61*(+, −) strain up to a total concentration of 900 µg/mL G418, with the G418 concentration increasing in 100 µg/mL intervals. Dilutions of the culture with 900 µg/mL G418 were plated on solid YM medium containing 900 µg/mL G418 to obtain single colonies, some of which were then selected. The genotype of the Δ*CYP61*(−, −) strain was confirmed by PCR using specific primers for the *CYP61* gene and the G418 resistant cassette (Fig. 3). Like the Δ*CYP61*(+, −) strain, the specific fragments of the G418 resistant cassette (C and D) and the theoretical length of fragment E from the G418 resistant cassette (4500 bp) were confirmed in the Δ*CYP61*(−, −) strain. In contrast, the specific fragments for *CYP61* (A and B) completely disappeared in the Δ*CYP61*(−, −) strain. These results indicated that both diploid *CYP61* genes were successfully deleted in the Δ*CYP61*(−, −) strain. Five Δ*CYP61*(−, −) strains were obtained from 60 colonies. Homologous recombination between the disrupted and non-disrupted allele regions was used to delete the *CYP61* gene from the Δ*CYP61*(+, −) strain. This was achieved by selection pressure applied through a gradual increase in antibiotic concentration.

### Ergosterol biosynthesis

Ergosterol biosynthesis in the parental host strain, Δ*CYP61*(+, −) and Δ*CYP61*(−, −) was evaluated by reversed phase high-performance liquid chromatography (RP-HPLC). Chromatograms of ergosterol extractions from the parental host strain, Δ*CYP61*(+, −) strain and Δ*CYP61*(−, −) strain are shown in Fig. 4a. In the parental host strain and Δ*CYP61*(+, −) strain, a peak at 280 nm at 15.6 min was observed and identified as the ergosterol peak, confirmed by the addition of standard ergosterol. Conversely, this ergosterol peak was not observed in the Δ*CYP61*(−, −) strain. As shown in the time course of ergosterol concentrations (Fig. 4b), ergosterol biosynthesis in the parental host strain and Δ*CYP61*(+, −) strain almost stopped after 24 h of cultivation. In contrast, ergosterol biosynthesis was not observed in the Δ*CYP61*(−, −) strain for more than 72 h from the start of the cultivation.

### Astaxanthin fermentation

**Fig. 5:**
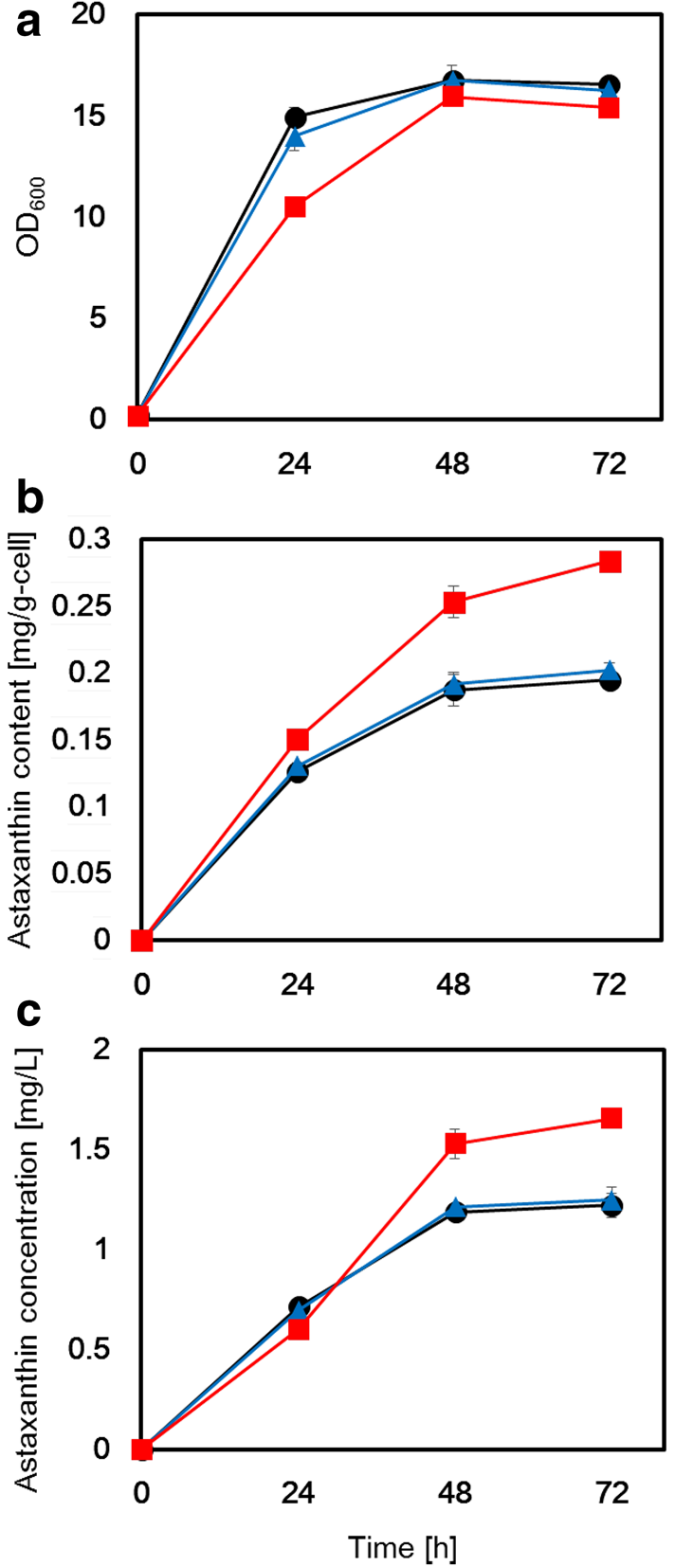
RP-HPLC ergosterol analysis from the parental host strain, Δ*CYP61*(+, −) strain and Δ*CYP61*(−, −) strain respectively, and astaxanthin production in each. **a** Cell concentration (OD_600_); **b** intracellular astaxanthin content (mg/g-cell). **c** volumetric astaxanthin concentration (mg/L). *Circle* (*black*), *triangle* (*blue*) and *square* (*red*) symbols represent values of the parental host strain, Δ*CYP61*(+, −) strain and Δ*CYP61*(−, −) strain, respectively

Astaxanthin production in the parental host strain, Δ*CYP61*(+, −) and Δ*CYP61*(−, −) was evaluated by RP-HPLC. Cell growth, intracellular astaxanthin content, and volumetric astaxanthin concentrations extracted from these strains are shown in Fig. 5. After 24 h of fermentation, cell growth rate had been obviously slower than that before 24 h of fermentation, even in the parental host strain (Fig. 5a), which would explain why additional ergosterol biosynthesis was not observed after 24 h (Fig. 4b). The cell concentration of the Δ*CYP61*(−, −) strain was lower than that of the parental host strain until after 48 h of fermentation while that of the Δ*CYP61*(+, −) strain was almost the same as the parental strain (Fig. 5a). Because ergosterol is a component of the cell membrane, this slower cell growth is caused by the suppression of ergosterol biosynthesis. In contrast, after 48 h of fermentation, the cell growth of all strains had stopped because glucose would be depleted.

Conversely, astaxanthin production decreased after 24 h of fermentation and was almost stopped after 48 h in the parental host strain and Δ*CYP61*(+, −) strain. In the Δ*CYP61*(−, −) strain, astaxanthin production instead continuously increased after 24 h of fermentation and gradually increased even after 48 h of fermentation (Fig. 5b). Finally, the volumetric astaxanthin concentration after 72 h of fermentation of the Δ*CYP61*(−, −) strain (1.65 mg/L) was approximately 1.4-fold higher than that of the parental host strain (1.22 mg/L), although that of Δ*CYP61*(−, −) strain (1.25 mg/L) was not changed (Fig. 5c). These results indicate that astaxanthin production was suppressed through feedback inhibition of the mevalonate pathway by ergosterol. The double deletion of diploid *CYP61* genes was critical to release this feedback inhibition, with the single disruption of *CYP61* having almost no effect on inhibition. The physiological concentration of ergosterol needed for feedback inhibition of the mevalonate pathway can be estimated to be 10-fold higher than the concentration of astaxanthin according to the results of Figs. 4b and 5c. The fold-change increase in astaxanthin production following *CYP61* gene deletion (1.6-fold) was almost equal to that (1.5-fold) caused by overexpression of genes involved in astaxanthin biosynthetic metabolism [8]. In contrast to these overexpression strategies that used wild-type strains as the host strain, a greater fold-change increase in astaxanthin production (3.4-fold) was observed when an astaxanthin-hyper producing mutant strain generated by random mutagenesis was used as a host strain [15]. However, the problem of random mutagenesis is reduced in the cell growth of the host strain compared with the wild-type strain [15] because unexpected mutations occurring through random mutagenesis, in addition to the expected mutations, result in enhanced astaxanthin biosynthesis [9, 10]. In contrast to random mutagenesis, the disruption of target genes carried out using the gene disruption method developed in this study did not decrease the final cell concentration because of the absence of any unexpected mutations. The developed strain has the potential to be one of the best strains for further improvement of astaxanthin production through metabolic engineering because it has an original metabolic background.

## Conclusion

The two previously described methods for deletion of target diploid genes in *X. dendrorhous* had significant room for improvement. In this study, we developed an easy, novel method for double deletion of target genes from the diploid genome of *X. dendrorhous* through a single genetic transformation event followed by cultivation with gradually increasing antibiotic concentrations. Compared with previous methods, this method allows for efficient deletion of target genes while saving antibiotic resistant markers for use in deleting or overexpressing additional target genes. This method makes it possible to obtain strains that have completely lost the target allele. Indeed, we deleted double diploid *CYP61* genes using this method and achieved improvement of astaxanthin production in *X. dendrorhous*. This method has the potential to improve industrial production of various isoprenoids in *X. dendrorhous* without a decrease in cell growth.

## Methods

### Strains and media

NovaBlue (Novagen, Madison, WI, USA) was used as the *E. coli* host strain for recombinant DNA manipulation. *X. dendrorhous* (NBRC 10129) was used as the parental host strain for gene expression. *E. coli* transformants were grown in Luria broth medium (10 g/L tryptone, 5 g/L yeast extract, and 5 g/L sodium chloride) supplemented with 100 µg/mL ampicillin. Transformants of *X. dendrorhous* were cultured in YM medium (5 g/L tryptone, 3 g/L yeast extract, 3 g/L malt extract and 10 g/L glucose). Yeast extract and malt extract were purchased from Becton–Dickinson (Sparks, MD, USA). Other chemicals were obtained from Nacalai Tesque (Kyoto, Japan) or Wako Chemicals (Osaka, Japan).

### Plasmid construction

Target genes were cloned by PCR using KOD-Plus-Neo DNA polymerase (Toyobo, Osaka, Japan). Nucleotide sequences of the cloning primers for target genes are shown in Additional file 1. To construct the G418 resistant *CYP61* disruption plasmid pKF-G418-g*CYP61*, the G418 resistance cassette was amplified by PCR from pKF-G418-AAT [8] using the P*gpd*-fw/T*gpd*-rv forward and reverse primer set. The 5′- and 3′-homologous regions of *CYP61* were also amplified by PCR from *X. dendrorhous* genomic DNA. The forward primer and reverse primer sets used for these amplifications were g-*CYP61*-5′-fw/g-*CYP61*-5′-rv and g-*CYP61*-3′-fw/g-*CYP61*-3′-rv, respectively. These fragments were connected by PCR using g-*CYP61*-5′-fw/g-*CYP61*-3′-rv, generating the *CYP61* disruption cassette. The length of the homologous sequence in the *CYP61* disruption cassette was designed to be 1000 base pairs. This cassette was cloned into the *Nde*I digested pKF-G418-AAT fragment, which included Amp^R^, using the In-Fusion^®^ HD Cloning Kit (Takara, Shiga, Japan), to construct pKF-G418-g*CYP61*.

### Yeast transformation

Transformation was carried out using the method described in previous reports [11, 16] with some modifications for the construction of competent cells as follows. *X. dendrorhous* parental strains were grown in 5 mL liquid YM medium at 22 °C with agitation at 250 rpm for 24 h. Sufficient volumes of each culture were inoculated into 150 mL liquid YM medium to achieve initial values of 0.03 optical density at 600 nm (OD_600_). Cultures were then grown at 22 °C with agitation at 120 rpm for 16.5 h. The *Nde*I digested plasmids pKF-G418-g*CYP61* were transformed into the parental *X. dendrorhous* host strain to construct the *CYP61* single disruption strain (Δ*CYP61*(+, −)).

### Double deletion of *CYP61* genes from genome in *X. dendrorhous*

The Δ*CYP61*(+, −) strain was cultivated in a test tube containing 5 mL of yeast mold (YM) media with 40 µg/mL of G418, incubated at 22 °C with agitation at 250 rpm for 24 h. A 200 µL aliquot of the culture was then inoculated into 5 mL YM medium plus 100 µg/mL of G418. After sufficient growth at 22 °C with agitation at 250 rpm, a 200 µL aliquot of the culture was inoculated into 5 mL YM medium plus 200 µg/mL G418. The sequential operations were carried out up to a total concentration of 900 µg/mL G418, with the G418 concentration increasing in 100 µg/mL intervals. Dilutions of the culture with 900 µg/mL G418 were plated on solid YM medium containing 900 µg/mL G418 to obtain single colonies, some of which were then selected. The genotype of the Δ*CYP61*(−, −) strain was confirmed by PCR using specific primers for the *CYP61* gene and the G418 resistant cassette.

### Astaxanthin fermentation of *X. dendrorhous* strains

*Xanthophyllomyces dendrorhous* strains with deleted target genes were grown in 5 mL liquid YM medium, also containing 40 µg/mL G418 as required, in test tubes at 22 °C with agitation at 250 rpm for 24 h. Sufficient volumes of each culture were inoculated into 80 mL liquid YM medium in a Sakaguchi flask to achieve initial OD_600_ values of 0.15. Cells were then grown at 22 °C with agitation at 120 rpm for less than 72 h.

### Extraction and RP-HPLC measurement of astaxanthin and ergosterol

Cell concentration was measured as OD_600_ after culturing for the appropriate time. The cell mass (dry cell weight) was calculated by 0.3786 × OD_600_. To measure the intracellular astaxanthin content of *X. dendrorhous* mutants, harvested cells were suspended in 1 mL acetone. The cells were broken using a bead shaker (Shake Master NEO, BMS, Tokyo, Japan) with zirconia beads. The cell extract was centrifuged at 8000×*g* at 4 °C for 10 min and the supernatant was then diluted with an appropriate volume of acetone for cover the range of following HPLC assay.

Astaxanthin concentration was determined using a HPLC machine (Shimadzu, Kyoto, Japan) equipped with a Develosil ODS-HG-5 column (Nomura Chemical, Aichi, Japan). The operating conditions were a temperature of 25 °C, with acetonitrile/methanol/2-propanol (85/10/5 [v/v]) as the mobile phase at a flow rate of 0.8 mL/min, and the detection was performed at 471 nm with a UV detector SPD-20A (Shimadzu).

Ergosterol concentration was also determined using a HPLC machine (Shimadzu) equipped with a Develosil ODS-HG-5 column (Nomura Chemical). The operating conditions were a temperate of 30 °C, with methanol/water (97/3 [v/v]) as the mobile phase at a flow rate of 1.0 mL/min, and the detection was performed at 280 nm with a UV detector SPD-20A (Shimadzu).

## Additional file

10.1186/s12934-016-0556-x DNA seuence of primers used in this study.

## Abbreviations

HMG-CoA: hydroxymethylglutaryl-coenzyme A
YM: yeast mold
PCR: polymerase chain reaction
RP-HPLC: reversed phase high-performance liquid chromatography
IPP: isopentenyl-pyrophosphate
DMAPP: dimethylallyl-pyrophosphate
FPP: farnesyl-pyrophosphate
GPP: geranyl-pyrophosphate
GGPP: geranylgeranyl-pyrophosphate

## References

[1] Vachali P, Bhosale P, Bernstein PS (2012). Microbial carotenoids. Methods Mol Biol.

[2] Muntendam R, Melillo E, Ryden A, Kayser O (2009). Perspectives and limits of engineering the isoprenoid metabolism in heterologous hosts. Appl Microbiol Biotechnol.

[3] Westfall PJ, Pitera DJ, Lenihan JR, Eng D, Woolard FX, Regentin R (2012). Production of amorphadiene in yeast, and its conversion to dihydroartemisinic acid, precursor to the antimalarial agent artemisinin. Proc Natl Acad Sci USA.

[4] Ajikumar PK, Xiao WH, Tyo KE, Wang Y, Simeon F, Leonard E (2010). Isoprenoid pathway optimization for Taxol precursor overproduction in *Escherichia coli*. Science.

[5] Melillo E, Setroikromo R, Quax WJ, Kayser O (2013). Production of α-cuprenene in *Xanthophyllomyces dendrorhous*: a step closer to a potent terpene biofactory. Microb Cell Fact.

[6] Rodríguez-Sáiz M, Godio RP, Alvarez V, de la Fuente JL, Martín JF, Barredo JL (2009). The NADP-dependent glutamate dehydrogenase gene from the astaxanthin producer *Xanthophyllomyces dendrorhous*: use of its promoter for controlled gene expression. Mol Biotechnol.

[7] Hara KY, Morita T, Endo Y, Mochizuki M, Araki M, Kondo A (2014). Evaluation and screening of efficient promoters to improve astaxanthin production in *Xanthophyllomyces dendrorhous*. Appl Microbiol Biotechnol.

[8] Hara KY, Morita T, Mochizuki M, Yamamoto K, Ogino C, Araki M (2014). Development of a multi-gene expression system in *Xanthophyllomyces dendrorhous*. Microb Cell Fact.

[9] Gassel S, Schewe H, Schmidt I, Schrader J, Sandmann G (2013). Multiple improvement of astaxanthin biosynthesis in *Xanthophyllomyces dendrorhous* by a combination of conventional mutagenesis and metabolic pathway engineering. Biotechnol Lett.

[10] Castelblanco-Matiz LM, Barbachano-Torres A, Ponce-Noyola T, Ramos-Valdivia AC, Cerda García-Rojas CM, Flores-Ortiz CM (2015). Carotenoid production and gene expression in an astaxanthin-overproducing *Xanthophyllomyces dendrorhous* mutant strain. Arch Microbiol.

[11] Mauricio N, Jennifer A, Salvador B, Dionisia S, Carla L, Marisela C (2008). Genomic organization of the structural genes controlling the astaxanthin biosynthesis pathway of *Xanthophyllomyces dendrorhous*. Biol Res.

[12] Jennifer A, Salvador B, Marisela C, Carla L, Andres M, Mauricio N (2008). Cloning of the cytochrome p450 reductase (*crtR*) gene and its involvement in the astaxanthin biosynthesis of *Xanthophyllomyces dendrorhous*. BMC Microbiol.

[13] Jennifer A, Ignacio R, Mauricio N, Dionisia S, Maria CR, Marcelo B (2014). Functional characterization of the *Xanthophyllomyces dendrorhous* farnesyl pyrophosphate synthase and geranylgeranyl pyrophosphate synthase encoding genes that are involved in the synthesis of isoprenoid precursors. PLoS ONE.

[14] Iris L, Maria SG, Salvador B, Dionisia S, Pilar M, Marcelo B (2012). Enhancement of carotenoid production by disrupting the C22-sterol desaturase gene (*CYP61*) in *Xanthophyllomyces dendrorhous*. BMC Microbiol.

[15] Gassel S, Breitenbach J, Sandmann G (2014). Genetic engineering of the complete carotenoid pathway towards enhanced astaxanthin formation in *Xanthophyllomyces dendrorhous* starting from a high-yield mutant. Appl Microbiol Biotechnol.

[16] Wery J, Gutker D, Renniers AC, Verdoes JC, van Ooyen AJ (1997). High copy number integration into the ribosomal DNA of the yeast *Phaffia rhodozyma*. Gene.

